# Differential regulation of jasmonate responses in multiple *jaz* mutants

**DOI:** 10.1080/15592324.2021.1997240

**Published:** 2021-11-01

**Authors:** Yue Zhang, Bei Liu, Jiaqi Zhai, Qinglei Wang, Susheng Song

**Affiliations:** a Beijing Key Laboratory of Plant Gene Resources and Biotechnology for Carbon Reduction and Environmental Improvement, College of Life Sciences, Capital Normal University, Beijing, China; b Hebei Key Laboratory of Soil Entomology, Cangzhou Academy of Agricultural and Forestry Sciences, Cangzhou, China

**Keywords:** Jasmonate, JAZs, growth, defense

## Abstract

The phytohormones jasmonates (JAs) regulate diverse aspects of plant growth and defense responses. The JA-ZIM domain (JAZ) family of repressors are targeted by the JA receptor Coronatine Insensitive 1 for ubiquitination and subsequent degradation via the 26S proteasome. We previously investigated the functions of JAZs in JA responses by analyzing *jaz* mutants of the phylogenetic group I (*jaz1/2/5/6*), group II/III (*jaz10/11/12*), group IV/V (*jaz3/4/7/9* and *jaz3/4/7/8/9*), and their high-order mutant *jaz1/2/3/4/5/6/7/9/10/11/12*. Here, we examined JA-regulated root growth, apical hook curvature, flowering time, and defense against the insect *Spodoptera exigua* in the intermediate *jaz* mutants *jaz1/2/5/6/10/11/12, jaz1/2/3/4/5/6/7/9*, and *jaz3/4/7/8/9/10/11/12*. This study shows that these *jaz* mutants differentially affect JA responses, suggesting the complexity of JA pathway in these multiple *jaz* mutants.

The phytohormones jasmonates (JAs) control plant growth and development, such as root growth, apical hook curvature, flowering, and stamen development, and affect plant resistance and defense responses against insect attacks.^1–5^ The JA-ZIM (JAZ) domain proteins function as core repressors of JA signaling and responses.^6–8^ In response to JA signals, the JAZ repressors are recruited and ubiquitinated by the JA receptor Coronatine Insensitive 1 (COI1), and are subsequently degraded by the 26S proteasome, leading to activation of JAZs-repressed transcriptional responses and JA responses.^6,7,9,10^

The thirteen Arabidopsis JAZ repressors are classed into phylogenetic group I (JAZ1/2/5/6), II (JAZ10), III (JAZ11/12), IV (JAZ3/4/9), and V (JAZ7/8/13).^6,7,11 –13^ The *jaz1–1/2-2/5-1/6-5* mutant of group I is partially insensitive to JA in root growth inhibition.^13^ The *jaz10–1/11–1/12–1* mutant of group II/III is hypersensitive to JA in root growth inhibition.^13^ *jaz1/2/5/6* and *jaz10/11/12* are more susceptible to the *Spodoptera exigua* larvae than wild-type.^13^ The *jaz3-5/4-1/7-1/9-1* and *jaz3-5/4-1/7-1/8-1/9-1* mutants of group IV/V flower later than wild-type.^13^ The high-order *jaz* mutants targeting most of JAZs, including *jaz1-1/2-2/3-5/4-1/5-1/6-5/7-1/9-1/10–1/11–1/12–1*,^13^ *jaz1-2*/*2–3*/*3-4*/*4-1*/*5–1*/*6–4*/*7–1*/*9–4*/*10–1*/*13–1* (*jazD*),^12^ and *jaz1-2*/*2–3*/*3–4*/*4–1*/*5– 1*/*6–4*/*7–1*/*8–V^2^*/*9–4*/*10–1*/*13–1* (*jazU*),^12^ are hypersensitive to JA-inhibitory root growth and apical hook curvature, hyposensitive to ethylene (ET)-enhanced apical hook curvature, more resistant to insect herbivores, and flower later than wild-type.^12,13^

To further understand JAZ family in JA responses, we performed genetic crosses with our previously characterized *jaz* mutants,^13^ and constructed intermediate *jaz* mutants, including *jaz1-1/2-2/5-1/6-5/10–1/11–1/12–1* of group I/II/III, *jaz1-1/2-2/3-5/4-1/5-1/6-5/7-1/9-1* targeting group I/IV/V(JAZ7), *jaz3-5/4-1/7-1/8-1/9-1/10-1/11-1/12-1* for group II/III/IV/V(JAZ7/8), and analyzed their JA responses.

The primary root growth of wild-type was inhibited by methyl-jasmonate (MeJA), and the undecuple *jaz1/2/3/4/5/6/7/9/10/11/12* mutant control exhibited shorter roots under either mock or MeJA treatment (Figure 1a).^13^ The primary root growth of *jaz1/2/5/6/10/11/12* and *jaz1/2/3/4/5/6/7/9* was similar to that of wild-type, while the root of *jaz3/4/7/8/9/10/11/12* was shorter than that of *jaz1/2/3/4/5/6/7/9/10/11/12* under mock or MeJA treatment (Figure 1a).

**Figure 1.**
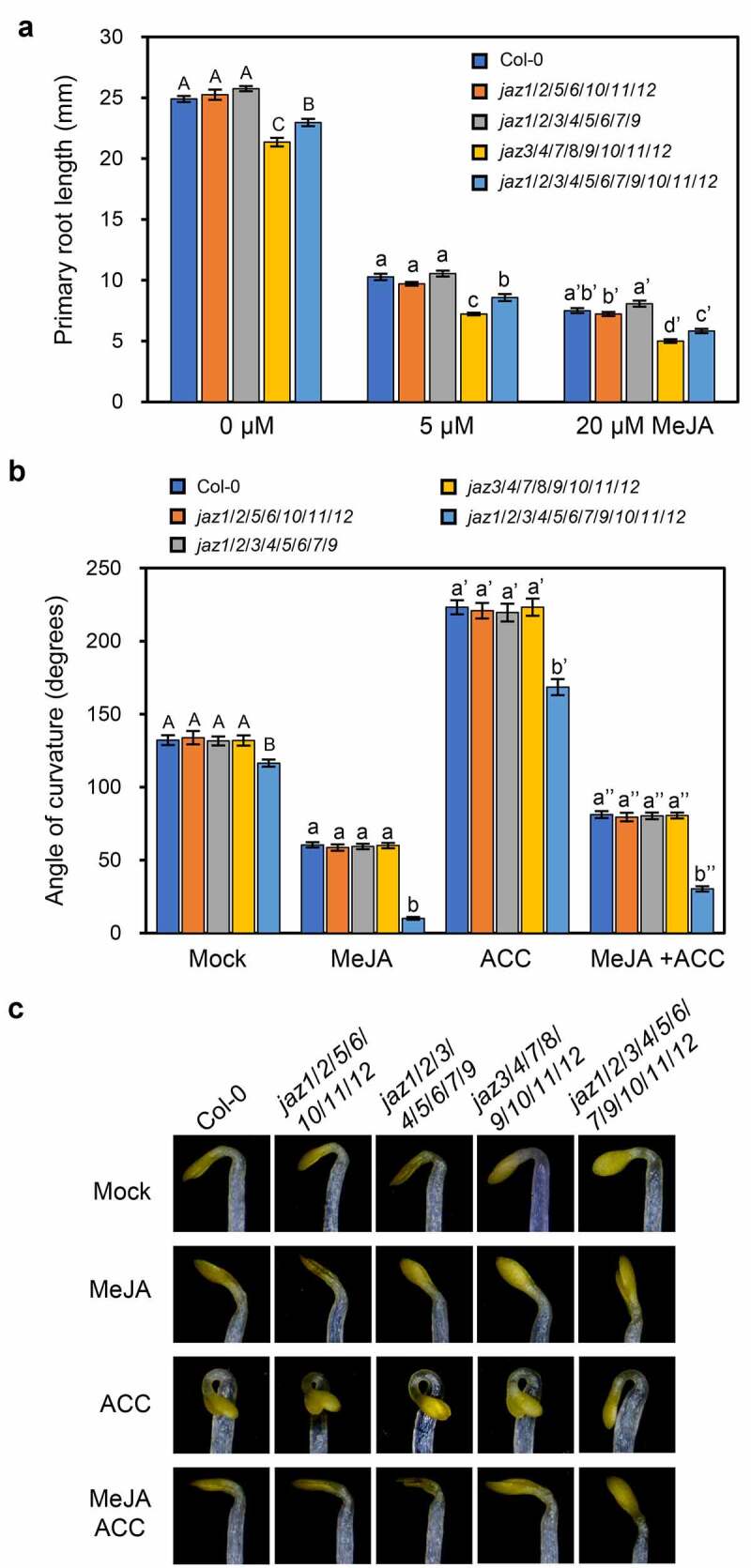
JA-regulated root growth and apical hook curvature in the multiple *jaz* mutants. (a) Primary root length of 11-day-old seedlings of the Col-0 wild-type, *jaz1*/*2*/*5*/*6*/*10*/*11*/*12, jaz1*/*2*/*3*/*4*/*5*/*6*/*7*/*9, jaz3*/*4*/*7*/*8*/*9*/*10*/*11*/*12*, and the *jaz1*/*2*/*3*/*4*/*5*/*6*/*7*/*9*/*10*/*11*/*12* control grown on MS medium containing 0, 5 or 20 μM MeJA under a 16-h (20–22°C)/8-h (18–20°C) light/dark photoperiod. Error bars represent SE (n = 20). (b, c) Apical hook curvatures (b) and hook phenotypes (c) of 4-day-old dark grown etiolated seedlings of the Col-0 wild-type, *jaz1*/*2*/*5*/*6*/*10*/*11*/*12, jaz1*/*2*/*3*/*4*/*5*/*6*/*7*/*9, jaz3*/*4*/*7*/*8*/*9*/*10*/*11*/*12*, and the *jaz1*/*2*/*3*/*4*/*5*/*6*/*7*/*9*/*10*/*11*/*12* control on MS medium supplied with mock, 5 μM MeJA, 10 μM ACC (1-aminocyclopropane-1-carboxylic acid, the ethylene biosynthesis precursor), or 5 μM MeJA plus 10 μM ACC. Data are means ±SE (n = 15). Letters indicate significant differences by Tukey’s HSD test (*P* < .05).

JA represses the apical hook curvature of etiolated *Arabidopsis* wild-type seedlings, while ET functions oppositely and antagonizes the JA action.^14^ The *jaz1/2/3/4/5/6/7/9/10/11/12* control displayed reduced hook curvature, was hypersensitive to JA-inhibited hook curvature and partially insensitive to ACC (ET precursor)-strengthened hook curvature (Figures 1b,Figures 1c).^13^ The *jaz1/2/5/6/10/11/12, jaz1/2/3/4/5/6/7/9* and *jaz3/4/7/8/9/10/11/12* mutants were indistinguishable from wild-type in JA/ET-regulated apical hook curvature (Figures 1b,Figures 1c), indicating the high redundancy of JAZ members in JA/ET-regulated hook curvature.

*jaz1/2/3/4/5/6/7/9/10/11/12* flowers later than wild-type, while *coi1-1* flowers earlier (Figures 2a,Figures 2b).^13^ Considering rosette leaf numbers at flowering or days to flowering (the appearance of a 1 cm-long bolt^15^), *jaz1/2/5/6/10/11/12* flowered earlier than wild-type (Figures 2a,Figures 2b). On the other hand, *jaz1/2/3/4/5/6/7/9* and *jaz3/4/7/8/9/10/11/12* flowered later than wild-type, but earlier than *jaz1/2/3/4/5/6/7/9/10/11/12* (Figures 2a,Figures 2b). *jaz1/2/3/4/5/6/7/9* and *jaz3/4/7/8/9/10/11/12* suppressed the early flowering phenotype of *coi1-1*, to the degree of *coi1-1 jaz1/2/3/4/5/6/7/9/10/11/12*, while *jaz1/2/5/6/10/11/12* did not obviously affect the flowering time of *coi1-1* (Figures 2c,Figures 2d).

**Figure 2.**
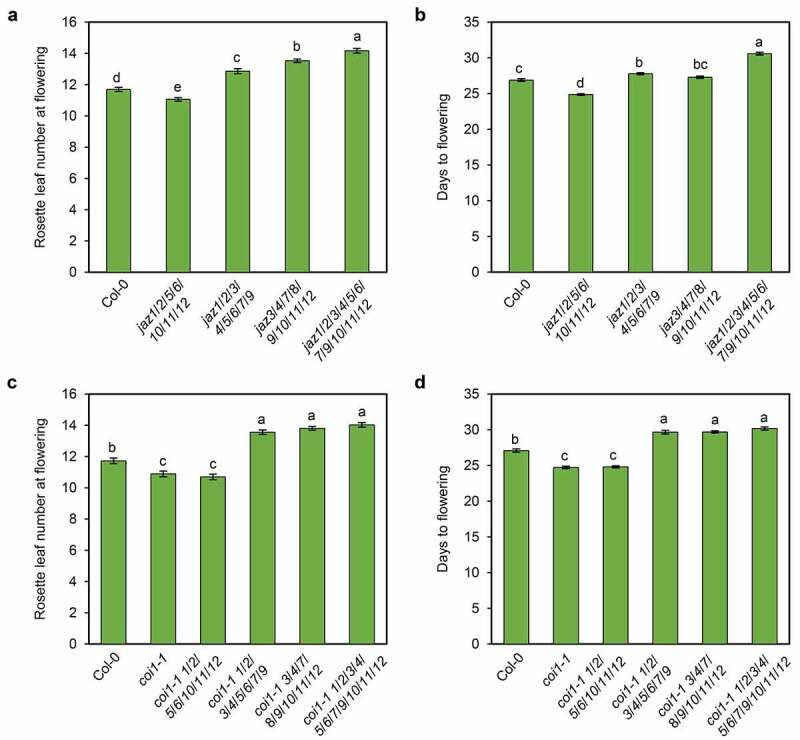
Flowering time in the multiple *jaz* mutants. Rosette leaf number at flowering (a, c) and days to flowering (b, d) of the Col-0 wild-type, *jaz1*/*2*/*3*/*4*/*5*/*6*/*7*/*9, jaz3*/*4*/*7*/*8*/*9*/*10*/*11*/*12*, and the *jaz1*/*2*/*3*/*4*/*5*/*6*/*7*/*9*/*10*/*11*/*12* control (a, b), or Col-0, *coi1-1*, and the indicated high-order *jaz* mutants in *coi1-1* background (c, d) grown under a 16-h (20–22°C)/8-h (18–20°C) light/dark photoperiod. Data are means ±SE (n = 36). Letters indicate significant differences by one-way ANOVA analysis with Tukey’s HSD post hoc test (*P* < .05).

*jaz1/2/3/4/5/6/7/9/10/11/12* is more resistant than wild-type in defense against the pest *Spodoptera exigua* (Figures 3a,Figures 3b).^13^The *S. exigua* larvae fed with *jaz1/2/3/4/5/6/7/9* and *jaz3/4/7/8/9/10/11/12* gained less weight than those with wild-type, but more weight than those with *jaz1/2/3/4/5/6/7/9/10/11/12* (Figures 3a,Figures 3b). On the other hand, *S. exigua* larvae reared with *jaz1/2/5/6/10/11/12* gained more weight than those with wild-type (Figures 3a,Figures 3b), suggesting that *jaz1/2/5/6/10/11/12* is more susceptible to *S. exigua* than wild-type.

**Figure 3.**
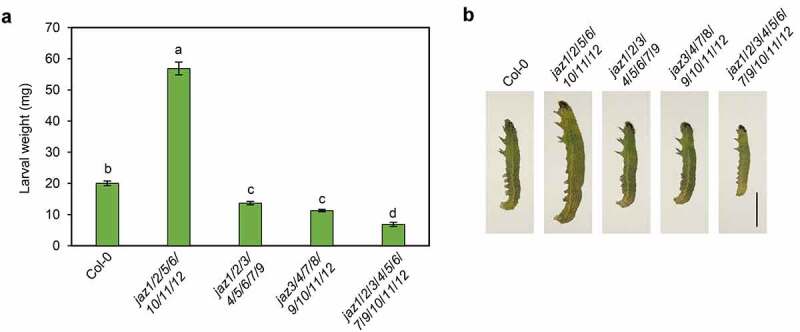
Multiple JAZs mutations differentially affect defense against insect attack. (a) and (b) Larval weights (a) and representative *S. exigua* larvae (b) after feeding for 14 days (a) or 7 days (b) on Col-0, *jaz1*/*2*/*3*/*4*/*5*/*6*/*7*/*9, jaz3*/*4*/*7*/*8*/*9*/*10*/*11*/*12*, and the *jaz1*/*2*/*3*/*4*/*5*/*6*/*7*/*9*/*10*/*11*/*12* control. 7-day-old seedlings grown under a 16-h (20–22°C)/8-h (18–20°C) light/dark photoperiod were transplanted into soil and grown under a 10-h (20–22°C)/14-h (18–20°C) light/dark photoperiod. The newly hatched *S. exigua* larvae were reared on 6-week-old plants. Error bars represent SE (n = 15). Scale bar = 0.2 cm. Letters indicate significant differences by one-way ANOVA analysis with Tukey’s HSD post hoc test (*P* < .05).

In conclusion, these results showed that the three high-order *jaz* mutants exhibited different effects on JA responses, including root growth, apical hook curvature, flowering time, and defense against insects, and indicate the redundancy of JAZ members and complexity of JA pathway in high-order *jaz* mutants.
